# Sex Differences in Hippocampal Memory and Kynurenic Acid Formation Following Acute Sleep Deprivation in Rats

**DOI:** 10.1038/s41598-018-25288-w

**Published:** 2018-05-03

**Authors:** Annalisa M. Baratta, Silas A. Buck, Austin D. Buchla, Carly B. Fabian, Shuo Chen, Jessica A. Mong, Ana Pocivavsek

**Affiliations:** 1 Maryland Psychiatric Research Center, Department of Psychiatry, University of Maryland School of Medicine, Baltimore, Maryland USA; 2 Division of Biostatistics and Bioinformatics, Department of Epidemiology and Public Health, University of Maryland School of Medicine, Baltimore, Maryland USA; 3 Department of Pharmacology, University of Maryland School of Medicine, Baltimore, Maryland USA

## Abstract

Inadequate sleep is a prevalent problem within our society that can result in cognitive dysfunction. Elevations in kynurenic acid (KYNA), a metabolite of the kynurenine pathway (KP) of tryptophan degradation known to impact cognition, in the brain may constitute a molecular link between sleep loss and cognitive impairment. To test this hypothesis, we investigated the impact of 6 hours of sleep deprivation on memory and KP metabolism (brain and plasma) in male and female rats. Sleep-deprived males were impaired in a contextual memory paradigm, and both sexes were impaired in a recognition memory paradigm. After sleep deprivation, hippocampal KYNA levels increased significantly only in males. The response in hippocampal KYNA levels to sleep loss was suppressed in gonadectomized males, delineating a role of circulating gonadal hormones. Circulating corticosterone, which has previously been linked to KP metabolism, correlated negatively with hippocampal KYNA in sleep-deprived females, however the relationship was not significant in male animals. Taken together, our study introduces striking sex differences in brain KYNA formation and circulating corticosterone in response to sleep deprivation. Relating these findings to sex differences in cognitive outcomes after sleep deprivation may further advance the development of novel therapeutic agents to overcome sleep loss-induced cognitive dysfunction.

## Introduction

Sleep loss and inadequate sleep are prevalent problems within our society^1^. Studies with both human and animal subjects have demonstrated that sufficient sleep is essential to protect mental health, physical health, and quality of life^2^. As cognitive dysfunction is one of the recurring consequences of sleep loss that impacts daily function, there is a compelling need to better understand the underlying molecular mechanism linking sleep loss and cognitive impairments. Our previous work demonstrates that kynurenic acid (KYNA), acting as an antagonist at *N*-methyl-d-aspartate (NMDA) and α7 nicotinic acetylcholine (α7nACh) receptors, is involved in cognitive processes in health and disease^3^. The current study presents evidence that KYNA may be a key player linking sleep loss and cognitive impairments.

**Figure 1:**
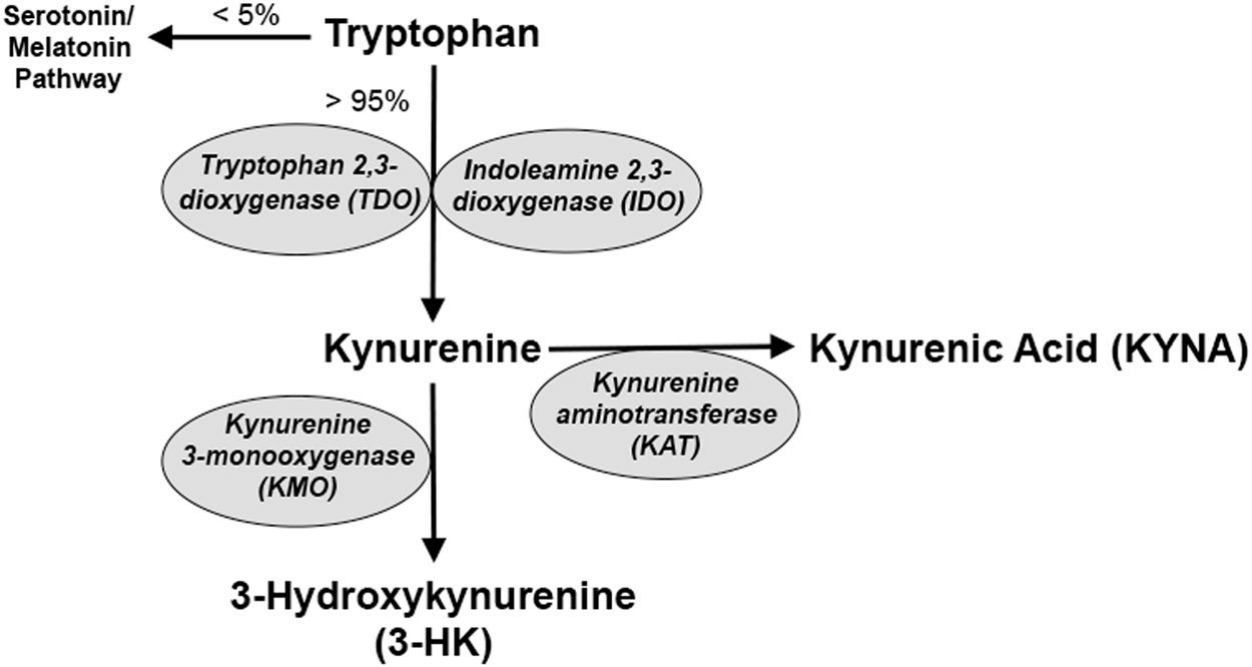
Schematic representation of the kynurenine pathway (KP) of tryptophan degradation.

KYNA is an endogenous astrocyte-derived neuroinhibitory molecule resulting from tryptophan degradation. Prolonged wakefulness has been shown to elevate tryptophan levels in the plasma and brain^4,5^. Tryptophan 2,3-dioxygenase (TDO) and indoleamine 2,3-dioxygenase (IDO) metabolize the vast majority of tryptophan to kynurenine and initiate the kynurenine pathway (KP; Fig. 1)^6^, while only about 5% of dietary tryptophan is degraded to serotonin and melatonin. Kynurenine promptly enters the brain from the blood, and is then degraded within local cells by kynurenine 3-monooxygenase (KMO) to 3-hydroxykynurenine (3-HK) or kynurenine aminotransferases (KATs) to KYNA.

The KP has remained relatively unexplored in sleep studies, however an association between sleep disturbances, excessive levels of KYNA, and cognitive impairments has been recently bolstered by studies in rats. Notably, increases in KP metabolism, including elevations in hippocampal KYNA, were found centrally and peripherally in a model of central fatigue induced by prolonged sleep deprivation in rodents^7,8^. Furthermore, we recently demonstrated that acute kynurenine treatment specifically reduces rapid eye movement (REM) sleep, REM-associated theta power, and impairs hippocampal-dependent contextual memory^9^.

To further investigate the hypothesized relationship between sleep and KYNA^7–9^, in designing our present studies we considered several important biological variables, including the impact of i) biological sex and circulating gonadal hormones and ii) sleep loss-induced stress. Of note, little emphasis has been placed on understanding sex differences in response to sleep deprivation. As sleep patterns^10^ and factors influencing KP metabolism may be sexually dimorphic^11–13^, for the present experiments we investigated the impacts of sleep deprivation in both male and female adult rats. Additionally, while the body adapts to sleep loss, several biological systems are activated, including the main neuroendocrine system regulating responses to stress, the hypothalamic-pituitary-adrenal (HPA) axis. During prolonged sleep deprivation, glucocorticoids, including cortisol in humans and corticosterone in rats, are released from the adrenal cortex and have been detected in plasma in both humans^14^ and rats^15^. As elevations in corticosterone may impact tryptophan metabolism via the KP^16^, we assess the relationship between KP activation and circulating corticosterone after sleep deprivation.

We demonstrated the effectiveness of our experimental paradigm, gentle handling, to induce sleep deprivation comparably in both sexes by acquiring polysomnographic recordings that combine electroencephalogram (EEG) and electromyogram (EMG). Interestingly, we determine a conspicuous sex difference in response to sleep deprivation in hippocampal-dependent contextual memory, but not recognition memory, which also relies on the perirhinal cortex. With sleep loss, male rats were significantly impaired in a passive avoidance paradigm and also showed increased hippocampal KYNA formation. To explore the mechanisms underlying the sex differences, the gonads were removed from adult male and female rats and biochemical assessments after sleep deprivation experiments were repeated. Taken together our data introduce a sex-dependent interplay between circulating gonadal hormones, the HPA axis activation, and KP metabolism in response to sleep deprivation.

## Materials and Methods

### Animals and experimental cohorts

Adult, male (200–300 grams; n = 102) and female (150–250 grams; n = 106) Wistar rats were obtained from Charles River Laboratories (Frederick, MD). Animals were housed in a temperature-controlled facility, fully accredited by the American Association for the Accreditation of Laboratory Animal Care, at the Maryland Psychiatric Research Center. They were kept on a 12/12 h light-dark cycle, where lights on corresponded to zeitgeber time (ZT) 0 and lights off to ZT 12, and received ad libitum access to food and water. All protocols were approved by the Institutional Animal Care and Use Committee at the University of Maryland School of Medicine and were in accordance with the National Institutes of Health *Guide for the Care and Use of Laboratory Animals*. All efforts were made to minimize animal suffering and to reduce the number of animals used. Experiment #1: animals underwent surgical procedures to implant telemetric transmitters to record sleep-wake behavior. Experiment #2: animals were used for behavioral testing. Experiment #3: tissue was collected from naïve animals after sleep deprivation. Experiment #4: tissue was collected from animals that underwent sham or gonad removal surgical procedures prior to sleep deprivation.

### Sleep deprivation protocol

Animals were sleep deprived for 6 h by gentle handling beginning at ZT 0. Individually housed animals remained in a home cage and were presented with novel objects (i.e. wooden blocks, paper towel, cotton balls) to induce exploration. When necessary, animals were gently touched with a long cotton tip applicator and cages were rotated to prevent the initiation of sleep.

### Sleep data collection and analysis

EEG and EMG recordings were conducted as described in previously published protocols^9^. Briefly, under isoflurane anesthesia, animals were placed in a David Kopf (Tujunga, CA) stereotaxic frame and implanted with telemetry transmitters (TL11M2-F40-EET; Data Science International, St. Paul, MN). EEG leads were wrapped around surgical screws (Plastics One, Roanoke, VA) inserted at 2.0 mm anterior/+1.5 mm lateral and 7.0 mm posterior/−1.5 mm lateral relative to bregma and secured with dental cement. EMG leads were inserted directly into the dorsal cervical neck muscle about 1.0 mm apart and sutured in place. Animals recovered for 10 days before experimentation.

All sleep data were recorded in a designated room. EEG and EMG waveform data were continuously collected using Ponemah 6.10 software (DSI). Digitized signal data were scored offline with Neuroscore 3.0 (DSI) in 10-s epochs as wake (low-amplitude, high-frequency EEG combined with high-amplitude EMG), non-REM (NREM)(high-amplitude, low-frequency EEG and low-amplitude EMG), or REM (low-amplitude, high-frequency EEG with very low EMG tone). The scored epochs were summed over 6 h during the light phase (ZT 0 to ZT 6). The total duration of time spent in each vigilance state was determined.

### Cognitive behavior assessment

#### Contextual memory task

The passive avoidance paradigm (PAP) was used to assess hippocampal-dependent contextual memory, as previously described^9^. Briefly, on day 1 (training trial) at ZT 3, animals were placed in the illuminated compartment of the test box and the latency to enter the dark compartment was recorded. A door separating the two compartments was immediately closed and an inescapable foot shock (0.56 mA for 1 s) was delivered through metal rods of the floor. Twenty-four hours later, at ZT 3 on day 2 (testing trial), the rat was again placed in the illuminated compartment, the guillotine door was opened, and the avoidance latency, i.e. the time to enter the dark compartment, was recorded.

#### Recognition memory task

The novel object recognition (NOR) task was used to assess recognition memory, engaging both the perirhinal cortex and hippocampus^17–19^. Briefly, on three consecutive days, rats were habituated for 5 min to the testing arena. Then, on the training day, each rat was given 5 min to explore two identical objects located in the arena. Twenty-four hours later, during the testing trial, each rat was again given 5 min to explore the arena which now contained one training trial object and one new object. The training and testing trial were video recorded and analyzed with ANY-Maze behavioral tracking software (Stoelting Co., Wood Dale, Illinois). Discrimination index was calculated as the difference in exploration time during the testing trial between novel object and familiar object, then dividing this value by the total object exploration time. The index takes into account individual differences in total exploratory behavior.

### Surgical procedures to remove gonads

All gonadectomy (GDX) surgeries were performed while animals were under isoflurane anesthesia. Briefly, in female animals, a small incision was made into the abdominal cavity from each flank. The ovary was withdrawn from the body, clamped and removed. The muscle wall was sutured and the incision was shut with wound clips. In male rats, a single incision was made above the scrotal sac. Each testis was withdrawn from the body cavity, clamped and removed. The incision was sutured closed. For each sex, sham surgeries were performed such that the described incisions were made, but the gonads were not removed. All animals recovered for at least 10 days before experimental testing.

### Tissue collection

At the conclusion of experiments, all animals were euthanized at ZT 6 by CO_2_ asphyxiation. Whole trunk blood was collected in tubes containing 20 μl K_3_-EDTA (0.15%). The blood was centrifuged (300 × g, 10 min) to separate plasma from cells and the supernatant was frozen at −80 °C. The brain was promptly removed and hippocampus and cortex were dissected. All regions were rapidly frozen on dry ice and stored at −80 °C.

### Biochemical analysis

#### Corticosterone measurement

Plasma corticosterone concentrations were determined by radioimmunoassay (MP Biomedicals, Solon, OH, USA). Samples were run in duplicate, with 5 μl of plasma used for each tube. The sensitivity threshold for the assay was 5 ng/mL and inter- and intra-assay coefficients of variance were less than 10%.

#### Plasma tryptophan, kynurenine, and KYNA determination

Plasma samples were diluted in ultrapure water (1:1,000 for tryptophan; 1:2 for kynurenine; 1:10 for KYNA). Twenty-five μl of 6% perchloric acid were added to 100 μl of the preparation. Precipitated proteins were separated by centrifugation (12,000 × g, 10 min) and analyzed by high performance liquid chromatography (HPLC). Twenty μl of the supernatant were applied to a ReproSil-Pur C18 column (4 × 150 mm; Dr. MaischGmbh, Ammerbuch, Germany), using a mobile phase containing 50 mM sodium acetate and 5% acetonitrile (pH adjusted to 6.2 with glacial acetic acid) at a flow rate of 0.5 ml/min, and detected with 500 mM zinc acetate delivered after the column with a flow rate of 0.1 ml/min. Tryptophan (excitation 285 nm, emission 365 nm), kynurenine (excitation 365 nm, emission 480 nm), and KYNA (excitation 344 nm, emission 398 nm) were detected fluorometrically in the eluate (Waters Alliance, 2475 fluorescence detector, Bedford, MA).

#### Brain KYNA determination

Hippocampal and cortical regions were weighed, sonicated in ultrapure water (1:10, w/v) and aliquoted. Twenty-five μl of 25% perchloric acid were used to acidify 100 μl of diluted sample. After centrifugation (12,000 × g, 10 min), 30 μl of the supernatant were subjected to HPLC as described above.

#### Brain 3-HK determination

Cortical samples were weighed, sonicated (1:5, w/v) in ultrapure water and aliquoted. Twenty-five μl of 6% perchloric acid were used to acidify 100 μl of diluted sample. After centrifugation (12,000 × g, 10 min), 20 μl of the supernatant was subjected to HPLC. 3-HK was eluted from a 3-μm HPLC column (HR-80; 80 × 4.6 mm; ESA) using a mobile phase of 1.5% acetonitrile, 0.9% triethylamine, and 0.59% phosphoric acid at a flow rate of 0.5 mL/min and detected electrochemically using an HTEC 500 detector (Eicom, San Diego, CA, USA; oxidation potential: + 0.5 V).

### Statistical analysis

#### Experiment #1

The percent of time spent in each vigilance state (REM, NREM, wake duration) was analyzed by repeated measures analysis of variance (ANOVA) comparing control and sleep deprivation. Bonferroni correction was used for multiple comparisons.

#### Experiment #2

Latency to enter the dark compartment of the PAP was analyzed by repeated measures ANOVA. The effects of sex and sleep were assessed. Exploration time in the NOR task was analyzed by repeated measures ANOVA, with object as a within subject variable. Main effects and interaction of sex and sleep were assessed between-subjects. The outcomes of NOR task discrimination index, distance traveled on the training day, and mean speed on the training day were analyzed by two-way ANOVA, with main effects and interaction of sex and sleep. Bonferroni correction was used for multiple comparisons.

#### Experiment #3

Biochemical analysis (plasma tryptophan, plasma kynurenine, plasma KYNA, cortical KYNA, cortical 3-HK, hippocampal KYNA, plasma corticosterone) of samples collected from naïve animals were analyzed by two-way ANOVA. For each analysis, main effects and the interaction of sex and sleep condition were assessed. Bonferroni correction was used for multiple comparisons.

#### Experiment #4

Biochemical analysis (plasma tryptophan, plasma kynurenine, plasma KYNA, hippocampal KYNA, plasma corticosterone) of samples collected from animals that underwent sham or gonadectomy surgery were analyzed by three-way ANOVA. For each analysis, sleep condition, sex, and surgery and possible interactions were assessed. The final model was selected by using a model selection procedure: when factors did not interact in three-way ANOVA (P > 0.05), analyses were followed up with appropriate two-way interactions; and analyses were focused on the main effects of sleep, sex, and surgery when two- way interactions were not statistically significant. Bonferroni correction was used for multiple comparisons.

In all analyses, where appropriate, significant main effects were followed up with the Bonferroni post hoc test. Spearman correlation analysis was performed to determine the relationship between peripheral corticosterone and hippocampal KYNA. All statistical analyses were performed using GraphPad Prism 6.0 (Graphpad Software, La Jolla, CA, USA) or SPSS 24 software (IBM Corporation, Armonk, USA). Statistical significance was defined as P < 0.05. See supplemental Fig. 1 for further details.

### Data Availability

The data generated in the present study are available from the corresponding author upon reasonable request.

## Results

### Gentle handling unequivocally disrupts sleep in male and female rats

**Figure 2:**
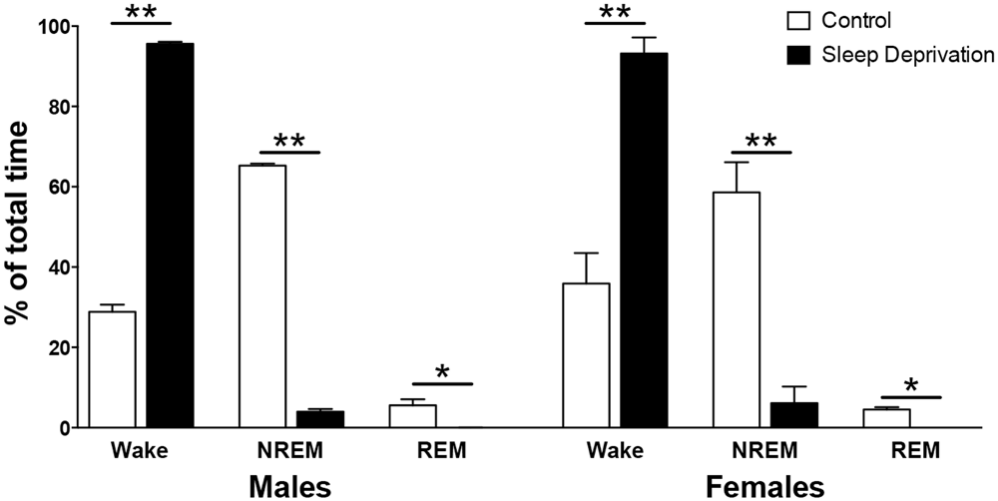
Gentle handling for 6 h from ZT 0 to ZT 6 effectively eliminates rapid eye movement (REM) and non-REM (NREM) sleep in male and female animals. All data are mean ± SEM. *P < 0.05, **P < 0.01. N = 3 per group.

Sleep deprivation by gentle handling from ZT 0 to ZT 6 resulted in 95% elimination of NREM and 100% elimination of REM sleep in male and female animals (Fig. 2).

### Acute sleep deprivation differentially disrupts memory in male and female rats

**Figure 3:**
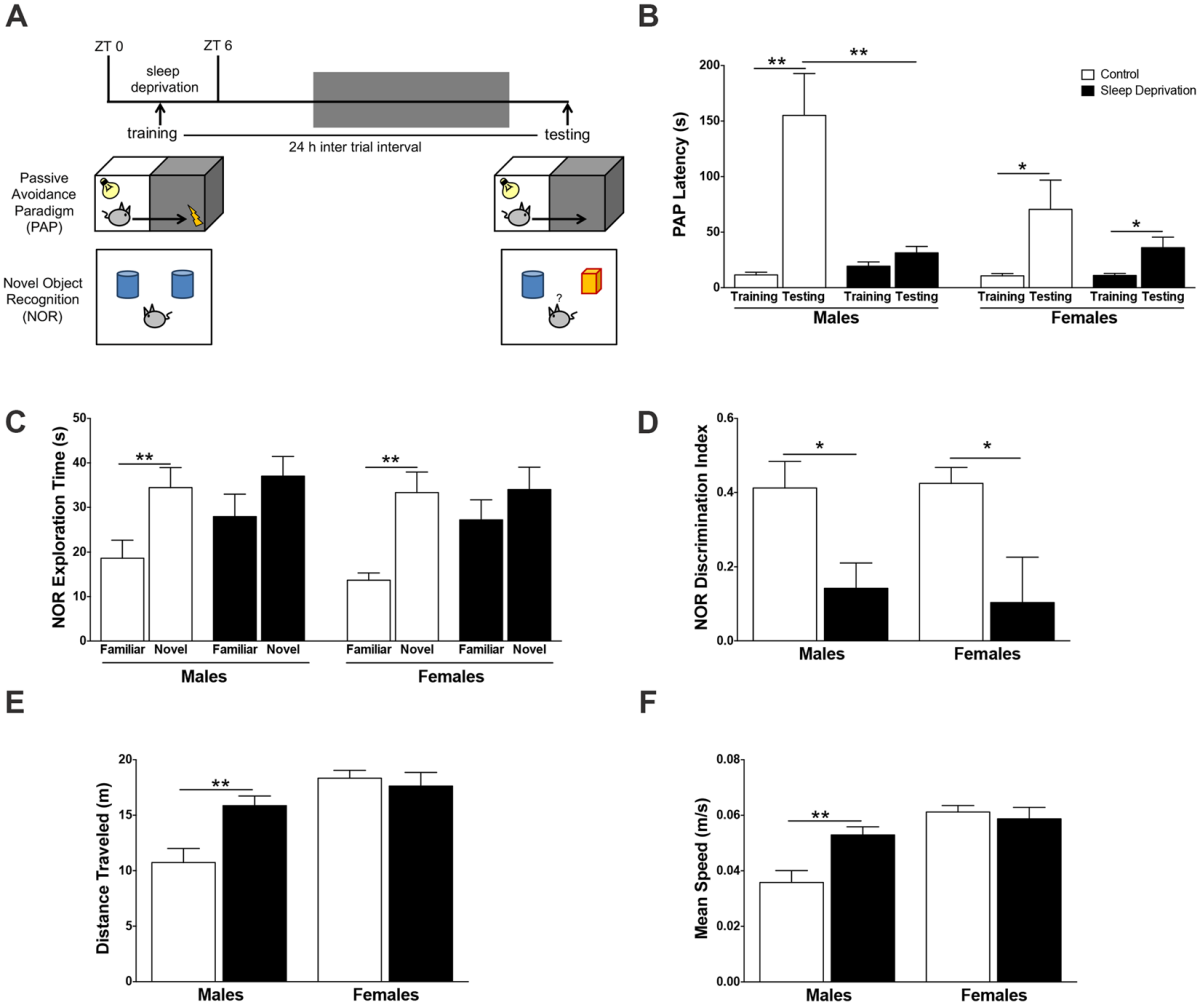
Sleep deprivation impairs both contextual and recognition memory in male rats, but only recognition memory in female rats. **(A)** Schematic representation of standard behavioral task to assess contextual memory, passive avoidance paradigm (PAP), and recognition memory, novel object recognition (NOR). Male and female animals were sleep deprived for 6 h from ZT 0 to ZT 6. **(B)** PAP Latency. **(C)** NOR exploration time during testing trial. **(D)** NOR discrimination index during testing trial. **(E)** Distance traveled during training trial of NOR. **(F)** Mean speed during training trail of NOR. All data are mean ± SEM. *P < 0.05, **P < 0.01. N = 8−12 per group.

We tested both sexes of rats in two behavioral tasks to assess learning and memory after sleep deprivation, the PAP to probe contextual memory and the NOR to evaluate recognition memory. As shown in Fig. 3A, animals were sleep deprived from ZT 0 to ZT 6 and underwent behavioral task training at ZT 3. Twenty-four hours after training, animals were assessed in the testing trial. Sleep condition significantly impacted the latency to enter the dark compartment on the test day of the PAP (F_1,37_ = 12.5, P < 0.01), while there were no significant effects of sex alone (F_1,37_ = 3.9, P = 0.1) or a sex x sleep condition interaction (F_1,37_ = 3.1, P = 0.09) (Fig. 3B). In male rats, sleep deprivation induced significant PAP deficits, evidenced as decreased avoidance latency on day 2 (P < 0.01). However, in female animals, compared to the control condition, the latency to enter the dark compartment was not significantly reduced on the test day with sleep loss (P = 0.40). Females that were sleep deprived displayed a significant increase in latency between the training and testing trial (P < 0.05), similar to control females (P < 0.05), thus indicating the ability to ordinarily learn in the PAP.

Both sexes of rats were significantly impaired in the NOR task after sleep deprivation. While control male and control female rats spent significantly more time with the novel object, sleep deprived animals failed to differentiate and spent equal time with the familiar and novel object during the test trial (Fig. 3C). Analysis of the discrimination index between objects revealed no significant sleep condition x sex interaction (F_1,41_ = 0.1, P = 0.75), but a significant main effect of sleep condition (F_1,41_ = 13.5, P < 0.0001) (Fig. 3D). Both male and female sleep deprived animals had a significantly lower discrimination index (P < 0.05) compared to counterpart controls. Of note, during the training trial in the NOR task, distance traveled during the task was significantly impacted by sex (F_1,41_ = 20.7, P < 0.0001), sleep condition (F_1,41_ = 4.6, P < 0.05) and an interaction between sex x sleep condition (F_1,41_ = 8.0, P < 0.01) (Fig. 3E). Additionally, the mean speed during the training trial was also impacted by sex (F_1,41_ = 20.2, P < 0.0001), sleep condition (F_1,41_ = 4.5, P < 0.05) and an interaction between sex x sleep condition (F_1,41_ = 7.9, P < 0.01) (Fig. 3F). Sleep-deprived males traveled a greater distance (P < 0.01) and also had an increase in mean speed (P < 0.01) in the testing apparatus on the training day, suggesting that loss of sleep induced a hyperactive state in males alone.

### No change to peripheral tryptophan, kynurenine, or KYNA after sleep deprivation

**Figure 4:**
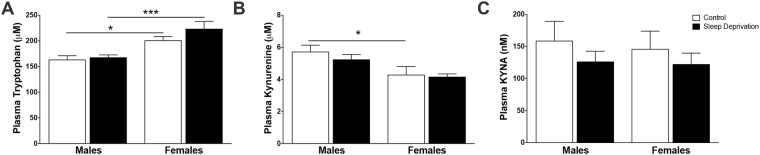
No change to peripheral tryptophan, kynurenine, or KYNA after sleep deprivation. Male and female animals were sleep deprived for 6 h from ZT 0 to ZT 6. **(A)** Plasma tryptophan. **(B)** Plasma kynurenine. **(C)** Plasma KYNA. All data are mean ± SEM. *P < 0.05, ***P < 0.001. N = 9 per group.

To examine the impact of acute sleep loss on the dynamics of KP metabolism, we determined tryptophan, kynurenine and KYNA levels in the plasma immediately after sleep deprivation in both sexes of rats. In the plasma, we found no significant effect of sleep condition on tryptophan (F_1,31_ = 1.8, P = 0.19) (Fig. 4A), kynurenine (F_1,32_ = 0.6, P = 0.44) (Fig. 4B) or KYNA (F_1,32_ = 1.4, P = 0.25) (Fig. 4C). Sex significantly impacted plasma tryptophan (F_1,31_ = 22.1, P < 0.001) and kynurenine (F_1,32_ = 10.6, P < 0.01). Post-hoc analysis revealed a significant difference between tryptophan and kynurenine among control male and female animals (P < 0.05) and tryptophan among sleep-deprived animals (P < 0.001).

### Sleep deprivation specifically impacts hippocampal KYNA in a sex-dependent manner

**Figure 5:**
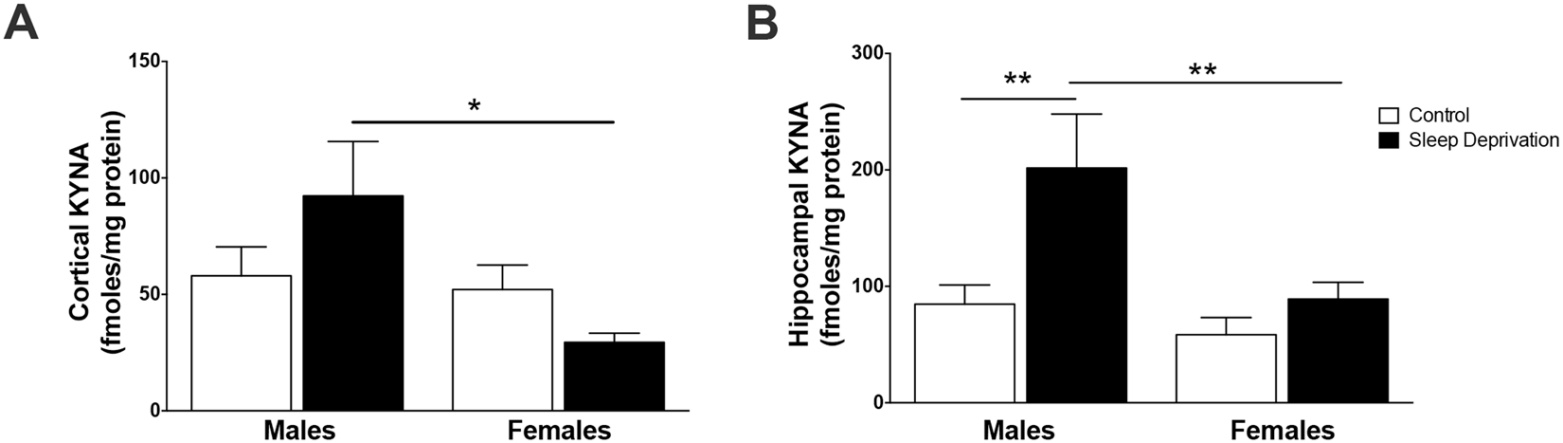
Elevated hippocampal KYNA in male, but not female, animals after sleep deprivation. Male and female animals were sleep deprived for 6 h from ZT 0 to ZT 6. **(A)** Cortical KYNA. **(B)** Hippocampal KYNA. All data are mean ± SEM. *P < 0.05, **P < 0.01. N = 7−9 per group.

Levels of KP metabolites KYNA and 3-HK were also assessed in the cortical tissue immediately after sleep deprivation. KYNA levels were significantly impacted by sex alone (F_1,29_ = 4.9, P < 0.05), but no main effect of sleep (F_1,29_ = 0.14, P = 0.7) or interaction (F_1,29_ = 3.4, P = 0.08) were found (Fig. 5A). Cortical KYNA levels were significantly less in sleep-deprived females than counterpart males (P < 0.05). Levels of 3-HK, the initial product of the alternative branch of kynurenine metabolism, were assessed in the cortical samples. 3-HK levels were not impacted by sleep deprivation (F_1,16_ = 0.21, P = 0.65), sex (F_1,16_ = 2.4, P = 0.14), and we found no significant sleep condition x sex interaction (F_1,16_ = 1.6, P = 0.22)(control male: 202 ± 13 fmoles/mg protein; sleep-deprived male: 179 ± 25 fmoles/mg protein; control female: 210 ± 38 fmoles/mg protein; sleep-deprived female: 260 ± 34 fmoles/mg protein).

Alterations in KYNA formation were also assessed in the hippocampus after sleep deprivation. KYNA in the hippocampus was impacted by both sleep deprivation (F_1,26_ = 8.4, P < 0.01) and sex (F_1,26_ = 7.4, P < 0.05) (Fig. 5B). However, there was no significant sleep condition x sex interaction (F_1,26_ = 2.9, P = 0.1). In male rats, there was a significant increase in hippocampal KYNA after sleep deprivation (P < 0.01). Within the sleep deprivation cohorts, females had significantly less hippocampal KYNA than male rats (P < 0.01).

### Impact of sleep deprivation on peripheral tryptophan, kynurenine, or KYNA in gonadectomized rats

**Figure 6:**
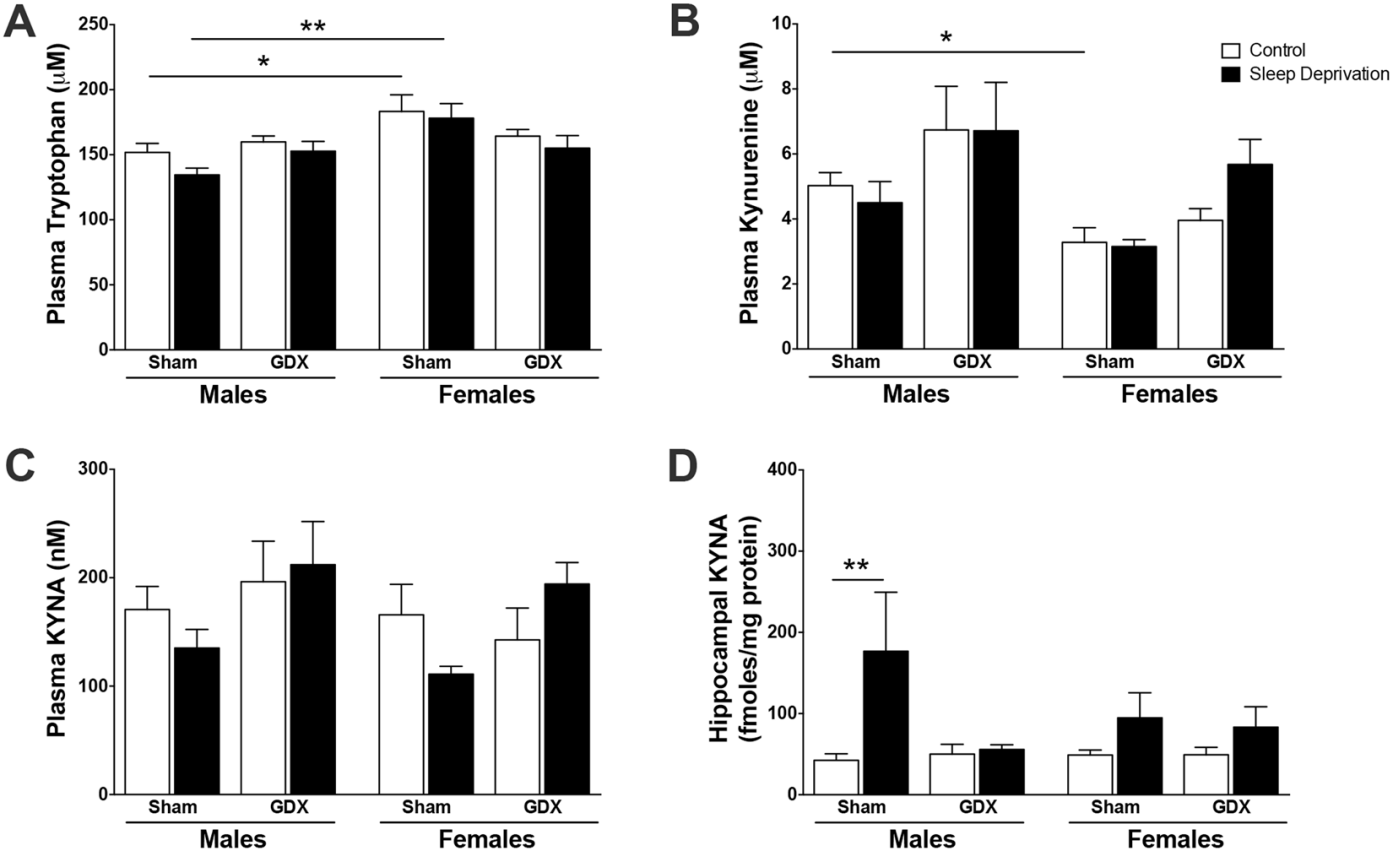
Gonadectomy (GDX) in male animals attenuates an elevation in hippocampal KYNA levels after sleep deprivation. Sham-operated or GDX male and female animals were sleep deprived for 6 h from ZT 0 to ZT 6. **(A)** Plasma tryptophan. **(B)** Plasma kynurenine. **(C)** Plasma KYNA. **(D)** Hippocampal KYNA. All data are mean ± SEM. *P < 0.05, **P < 0.01. N = 5–11 per group.

To decipher if our sex-specific findings were mediated by circulating gonadal hormones, we next assessed KP biochemical changes in gonadectomized rats. Tryptophan in the plasma was not significantly affected by a three-way sleep condition x sex x gonadectomy surgery interaction (F_1,70_ = 1.0, P = 0.3) (Fig. 6A). Two-way analysis revealed a significant sex x surgery interaction (F_1,70_ = 7.5, P < 0.01), but no impact of sleep condition x surgery (F_1,70_ = 0.01, P = 0.93) or sleep condition x sex (F_1,70_ = 0.7, P = 0.4). As in the naïve condition (see Fig. 4A), plasma tryptophan was significantly impacted by sex (F_1,70_ = 11.2, P < 0.01), however there were no main effects of sleep condition (F_1,70_ = 2.0, P = 0.16) or surgery (F_1,70_ = 0.13, P = 0.72). Plasma tryptophan was elevated in sham control and sleep-deprived females compared to sham control and sleep-deprived males, as we previously determined.

Plasma kynurenine was not impacted by a three-way sleep condition x sex x surgery interaction (F_1,53_ = 0.3, P = 0.59) (Fig. 6B). Additionally, no significant two-way interactions were determined (sleep x sex: F_1,53_ = 0.7, P = 0.39; sleep x surgery: F_1,53_ = 0.9, P = 0.35; sex x surgery: F_1,53_ = 0.1, P = 0.77). While there was no main effect of sleep (F_1,53_ = 0.2, P = 0.68), plasma kynurenine was significantly impacted by sex (F_1,53_ = 7.8, P < 0.01) and surgery (F_1,53_ = 8.3, P < 0.01) alone. Post-hoc analysis revealed significantly reduced plasma kynurenine in sham control females compared to sham control males, as we previously determined.

When plasma KYNA levels were assayed, no three-way interaction between sleep x sex x surgery was determined (F_1,72_ = 0.2, P = 0.68) (Fig. 6C). Additionally, no significant two-way interactions were found (sleep x sex: F_1,72_ = 0.03, P = 0.86; sleep x surgery: F_1,72_ = 1.4, P = 0.24; sex x surgery: F_1,72_ = 0.4, P = 0.52). Sleep condition (F_1,72_ = 0.002, P = 0.96) and sex (F_1,72_ = 0.2, P = 0.67) had no main effect, but notably surgery significantly impacted plasma KYNA (F_1,72_ = 4.7, P < 0.05). There were no significant post-hoc findings.

### Male gonadectomy attenuates hippocampal KYNA elevation in response to sleep deprivation

Next, we assessed hippocampal KYNA levels in response to sleep deprivation in gonadectomized rats (Fig. 6D). No three-way interaction between sleep condition, sex, and surgery (F_1,63_ = 2.1, P = 0.15) was determined for hippocampal KYNA. Additionally, no significant two-way interactions were determined (sleep x sex: F_1,63_ = 0.6, P = 0.46; sleep x surgery: F_1,63_ = 3.0, P = 0.09; sex x surgery: F_1,63_ = 1.6, P = 0.46). There was a significant main effect of sleep condition (F_1,63_ = 7.4, P < 0.01), but no main effect of sex (F_1,63_ = 0.4, P = 0.55) or surgery (F_1,63_ = 2.4, P = 0.13). Sham sleep-deprived males had significantly more hippocampal KYNA than sham control males, but KYNA levels were not significantly different between control GDX males and sleep-deprived GDX males. KYNA remained unchanged after sleep deprivation in both sham and GDX females.

### Sleep deprivation by gentle handling dimorphically elevates plasma corticosterone in male and female rats

**Figure 7:**
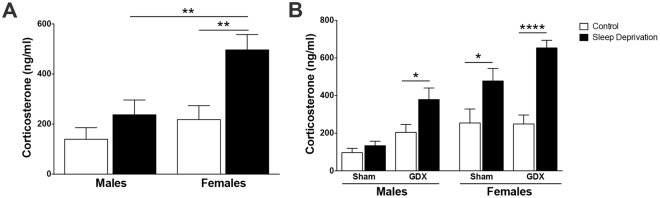
Sleep deprivation elevates corticosterone in female, but not male, animals. Plasma corticosterone in **(A)** intact or **(B)** sham-operated or GDX male and female animals that were sleep deprived for 6 h from ZT 0 to ZT 6. All data are mean ± SEM. *P < 0.05, **P < 0.01, ****P < 0.0001. N = 8−11 per group.

Due to the distinct behavioral and KP biochemical outcomes in male and female rats, we examined the stress response hormone corticosterone in the plasma after sleep deprivation. In the intact cohort, plasma corticosterone was significantly impacted by sleep condition (F_1,32_ = 11.4, P < 0.01) and sex (F_1,32_ = 9.2, P < 0.01) (Fig. 7A). Corticosterone was elevated by 130% in sleep-deprived females compared to control females. Male animals, however, did not have significantly higher corticosterone after sleep deprivation.

To determine whether gonadal hormones play a role in the observed differential response in circulating corticosterone, sleep deprivation experiments were conducted in the gonadectomized cohort of adult male and female rats. Plasma corticosterone was not impacted by a three-way sleep condition x sex x surgery interaction (F_1,72_ = 0.095, P = 0.76) (Fig. 7B). There was a significant interaction between sleep condition and sex (F_1,72_ = 8.8, P < 0.01), sleep condition and surgery (F_1,72_ = 5.2, P < 0.05), but no significant interaction between sex and surgery (F_1,72_ = 1.7, P = 0.20). Analysis of main effects determined that plasma corticosterone was impacted significantly by sleep (F_1,72_ = 36.1, P < 0.001), sex (F_1,72_ = 34.4, P < 0.001), and surgery (F_1,72_ = 14.0, P < 0.001). Sleep-deprived GDX males had significantly more plasma corticosterone than control GDX males (P < 0.01), while corticosterone was not elevated in response to sleep deprivation in sham-operated males (P = 0.53). Sleep deprivation significantly increased plasma corticosterone in both GDX females (P < 0.001) and sham-operated females (P < 0.05), compared to respective controls.

### Correlational analysis

Given that across multiple experiments we determined sexually differentiated responses in peripheral corticosterone and hippocampal KYNA elevation after sleep deprivation, we assessed a relationship between these two factors. No relationship between plasma corticosterone and hippocampal KYNA in intact control males (r = 0.03, P = 0.91) and intact control females (r = −0.21, P = 0.46) was found. In addition, while no relationship between corticosterone and hippocampal KYNA was determined in intact sleep-deprived males (r = 0.48, P = 0.07), interestingly, a significant negative correlation was found in intact sleep-deprived females (r = −0.44, P < 0.05). Taken together, these data suggest a sex-specific relationship between brain KYNA and peripheral corticosterone in response to sleep deprivation.

## Discussion

The results of our present study, which was designed to assess the short-term impact of sleep deprivation on KP metabolism, revealed several intriguing phenomena and sexually differentiated responses. We determined subtle sex differences in behavioral outcomes after sleep deprivation and found an elevation in hippocampal KYNA, specifically in male animals, in response to sleep deprivation. While female animals did not present significantly elevated brain KYNA, we report a female-specific elevation in circulating corticosterone, demonstrating a differential response in HPA reactivity after acute sleep loss. Taken together, our findings yield an unexpected sex difference and a selective impact of sleep deprivation on the KYNA branch of kynurenine metabolism in the brain.

In experimental animals, sleep deprivation has been induced by various techniques, including the platform over water method, which selectively deprives animals of paradoxical sleep or gentle handling, which is very effective at inducing total sleep deprivation and strongly models sleep loss in humans^20^. While prolonged methods of sleep deprivation activate sleep loss-induced stress, the HPA axis, and influence several biological systems^21^, studies conducted mostly in male animals have suggested that gentle handling does not activate the HPA axis and serves as a useful tool to delineate impacts of stress and sleep loss^20^. In our first experiments, we verified the efficacy of gentle handling as a method of sleep deprivation and determined unequivocal elimination of NREM (95%) and REM (100%) sleep in male and female rats. Of note, corticosterone levels were significantly higher only in the female sleep-deprived group immediately after sleep loss, suggesting prompt stress reactivity in females. Several studies have indicated that male rodents habituate more efficiently to stressors than female rodents^22^, providing a feasible explanation for the differential corticosterone response to sleep deprivation by gentle handling. Additionally, testosterone has been shown to inhibit glucocorticoid activation^23,24^, supporting our findings of potentiated corticosterone elevation in response to sleep deprivation in GDX males that lacked circulating testosterone. Circulating estradiol in females may account for their susceptibility to elevations in corticosterone^22^, but our subsequent experiment in GDX females challenges the notion that estrogens alone contribute to the phenomena in females. In the future, investigations involving gonad removal with sex hormone replacement will further clarify the role of the circulating hormones on HPA axis activation after sleep deprivation.

The fact that female rats were shielded from impairments in contextual memory as well as an elevation in brain KYNA after sleep deprivation deserves special consideration. Perhaps in part due to growing evidence that alterations in female circulating hormones influence various sleep-wake parameters^10^, the majority of sleep-wake studies have focused on male animals and our understanding of the impacts of sleep disruptions between sexes remains relatively elusive. In this context, we were outwardly surprised to find such striking sex differences, particularly because prolonged sleep deprivation in female rats alone has been shown to impair cognitive function^25,26^ and elevate cerebral KP metabolism^7,8^.

The implications of elevated KYNA after acute sleep loss in the hippocampus, which mediates learning and memory and is particularly compromised by sleep loss^27,28^, deserves discussion. Herein, we demonstrate functionally detrimental levels of hippocampal KYNA^3^ in intact males after sleep loss. Only the male animals in our studies were compromised cognitively by sleep deprivation in both the PAP and NOR tasks. These tasks were chosen based on several considerations, including i) the ability to train rats in a single trial and test 24 hours later and ii) the participation of the hippocampus in the execution of both tasks^19,29,30^. Our findings suggest a resilient advantage of female animals to acute sleep loss in the PAP task, but not the NOR task. Importantly, the NOR task relies on an interaction between the perirhinal cortex and hippocampus to engage recognition memory, particularly, as in our study design, when the inter trial delay is greater than 10 min^19,31^. To further understand the role of KYNA in mediating the relationship between sleep loss and recognition memory deficits, KYNA formation in the perirhinal cortex will be assessed in future experiments. In addition, we report sex differences in response to sleep deprivation in locomotor activity during the NOR training trial. Our data show that during the sleep deprivation period, males were hyperactive, evidenced as moving a greater distance and also at a greater speed. Taken together, while the underlying mechanisms governing these behaviors and the interplay of sex hormones remain to be elucidated, similar sex differences in cognitive performance are speculated in human studies wherein physiological responses to sleep deprivation are not equal among women and men^32,33^.

In rodents, sleep deprivation studies have focused extensively on narrow time windows of memory consolidation and identified that memory appears most sensitive to sleep loss after acquisition. Recovery from sleep deprivation, termed rebound sleep, is regulated by both a homeostatic and circadian drive^34^. This rebound sleep is characteristically more intense and promotes performance recovery in cognitive domains impacted by sleep loss^35^. An increase in KYNA in the hippocampus during this rebound sleep may be particularly detrimental for memory consolidation processes. In rodents, nanomolar and low micromolar concentrations of KYNA itself, or stimulation of KYNA synthesis with the direct bioprecursor kynurenine, causes a spectrum of cognitive deficits, including disruptions in hippocampus-mediated contextual learning and memory^3,36,37^. Through its inhibitory actions at α7nACh and NMDA receptors, KYNA has been hypothesized to impact cognition. In addition, we also recently demonstrated that acute elevations in KYNA disrupt sleep-wake architecture^9^, introducing a novel physiological role for KYNA and taken together with our present findings, we speculate that an increase in KYNA after sleep deprivation may negatively impact rebound sleep and contribute to impairments in memory consolidation. Thus, we predict that inhibition of KYNA synthesis may serve as an efficacious strategy to overcome sleep-loss induced memory problems, as decreases in brain KYNA levels have been associated with pro-cognitive benefits^3,38^. However, it certainly remains to be seen if this treatment strategy would be only effective in male animals.

We chose to consider KP metabolism and elevated KYNA as a possible outcome after sleep deprivation based on several considerations. As an essential amino acid and precursor to serotonin and melatonin, tryptophan has been extensively studied as a modulator of sleep and its accumulation in the plasma and brain has been confirmed after sleep deprivation^4,5^. The bioavailability of tryptophan can directly influence kynurenine and KYNA formation. When developing a rodent model of chronic fatigue, Yamashita and Yamamoto demonstrated that several days of sleep loss in rats activates the KP by elevating blood tryptophan and kynurenine, as well as enhancing kynurenine and KYNA formation in specific brain regions, including the hippocampus^7,8^. As free forms of tryptophan and kynurenine readily enter the brain, it is plausible that an activation of KYNA formation is due to an increase in bioprecursor availability. Presently, analysis of tryptophan, kynurenine, and KYNA levels demonstrated no significant alterations after acute sleep deprivation, despite hippocampal elevations in KYNA levels in intact male animals. Our findings suggest a brain-specific mechanism for these alterations after an acute episode of sleep deprivation. While several methodological details differ between our studies, namely method of deprivation and total duration of sleep loss, our findings concur that KYNA formation is enhanced in the hippocampus after sleep deprivation, and we synergistically support the notion that these elevations may have detrimental impacts on cognition.

Mechanistically several factors that influence KYNA synthesis in the brain may be altered after sleep deprivation. From the blood, kynurenine is rapidly accumulated in astrocytes and irreversibly transaminated by several KATs, including KAT II, to KYNA^3^. Sleep deprivation can impact processes that regulate KAT II activity, such as alterations in energy metabolism or bioavailability of pyruvate^39,40^, a co-substrate of the KAT reaction^41^. Alternatively, changes in KMO activity and subsequent 3-HK formation can also impact KYNA levels^42^. As 3-HK levels were not altered after sleep deprivation, variations in KMO activity may not be the contributing factor, but follow-up studies are necessary to further elucidate these dynamics.

Increases in kynurenine levels, or activity of the catabolic enzymes, are not the only mechanisms that determine the formation of KYNA in the brain. Alternatively, accumulation of pro-oxidant factors after sleep deprivation^43^ may promote non-enzymatic degradation of kynurenine to KYNA^44,45^. In addition, accumulating literature implicates astrocytes and gliotransmitters in modulating sleep pressure. After prolonged wakefulness, a buildup of adenosine^46^ may stimulate KYNA production, as suggested in a microdialysis study wherein local brain infusions of adenosine or an A_1_ receptor agonist elevated extracellular KYNA^47^. Lastly, as degradative enzymes for KYNA are not present in the mammalian brain^48^, and the metabolite has strikingly stable chemical properties, no specific processes are known to break down KYNA. Extracellular KYNA must compete with other acidic compounds endogenous to the brain for reuptake by organic anion transporters^49^ or be removed from the cerebrum by probenecid-sensitive process^50^, which may be impacted by periods of sleep loss. It remains to be seen, however, the exact mechanism by which KYNA levels fluctuate in the brain upon sleep loss.

In rats, physiological stressors have been shown to induce tryptophan metabolism, resulting in increased levels of kynurenine and downstream KP metabolites both in the brain and periphery^16,51,52^. Stimulation of the enzymes TDO and/or IDO, by elevations in corticosterone or immunological activation that occurs with stress reactivity respectively^16,53^, is thought to activate KP metabolism by promoting the catabolism of tryptophan. These effects could also play a role in enhanced inflammation with sleep-loss^54^, and contribute to misbalance in the KP in health and disease processes^12,55^. Importantly, our present data draw additional attention to sex as an important biological variable in KP studies^12,13,56,57^, and introduce a novel interplay between the HPA responses to sleep deprivation and downstream KP metabolism. The specific mechanisms governing the causes and physiological ramifications of these responses will be further explored and clarified in future studies.

Lastly, distinct abnormalities in tryptophan metabolism via KP are found in several psychiatric and neurological conditions^58^ that exhibit cognitive dysfunction and sleep disturbances as core symptoms. Sleep disturbances in patients with schizophrenia and depression may aggravate the severity of the disorders and often negatively impact the patients’ quality of life^59^. Conceivable hypotheses suggest that cognitive impairments and abnormalities in sleep are connected, and the potential contribution of KYNA in mechanistically mediating a relationship between sleep and cognition in schizophrenia patients remains under investigation^55,60^.

In conclusion, the present study demonstrates a significant elevation in brain KYNA levels after acute sleep deprivation in male animals, supporting the notion that KYNA serves as a key molecular link between sleep disturbances and cognitive dysfunction. Defining a causal relationship between sleep disturbances and KP metabolism may unravel KYNA as a novel nexus between sleep and cognition.

## Electronic supplementary material

Supplemental Table 1
