# Supplementary material for: Sex Differences in Hippocampal Memory and Kynurenic Acid Formation Following Acute Sleep Deprivation in Rats
Source: Sci Rep. 2018 May 3;8:6963. doi: 10.1038/s41598-018-25288-w (PMC5934413; doi:10.1038/s41598-018-25288-w)
Supplement: Supplementary file 1 — Supplemental Table 1 [file 41598_2018_25288_MOESM1_ESM.pdf]

Sex Differences in Hippocampal Memory and Kynurenic Acid Formation Following Acute Sleep Deprivation in Rats

Annalisa M. Baratta[1], Silas A. Buck[1], Austin D. Buchla[1], Carly B. Fabian[1], Shuo Chen[1,2]

Jessica A. Mong[3], Ana Pocivavsek[1*]

[1]Maryland Psychiatric Research Center, Department of Psychiatry, University of Maryland School of Medicine, Baltimore, Maryland (USA)

[2]Division of Biostatistics and Bioinformatics, Department of Epidemiology and Public Health, University of Maryland School of Medicine, Baltimore, Maryland (USA)

[3]Department of Pharmacology, University of Maryland School of Medicine, Baltimore, Maryland (USA)

Correspondence:
Ana Pocivavsek, Ph.D.
Maryland Psychiatric Research Center
P.O. Box 21247
Baltimore, Maryland 21228 (USA)
Phone: (410) 402-7319; Fax: (410) 747-2434
apocivavsek@som.umaryland.edu

Data	Figure	Statistical test	Interaction	Significance	Main Effect	Significance	Bonferroni post hoc test
Wake vigilance state	2	2-way repeated measures ANOVA	sex x sleep	P = 0.47	sleep	P < 0.001	C male vs SD male P < 0.01<br>C female vs SD female P < 0.01
					sex	P = 0.31	
NREM vigilance state	2	2-way repeated measures ANOVA	sex x sleep	P = 0.49	sleep	P < 0.001	C male vs SD male P < 0.01<br>C female vs SD female P < 0.01
					sex	P = 0.26	
REM vigilance state	2	2-way repeated measures ANOVA	sex x sleep	P = 0.57	sleep	P < 0.01	C male vs SD male P < 0.05<br>C female vs SD female P < 0.05
					sex	P = 0.54	
PAP latency	3B	3-way repeated measures ANOVA within subject	day x sex x sleep	P = 0.05	day	P < 0.0001	C male P < 0.01;<br>C female P < 0.05;<br>SD female P < 0.05
			day x sex	P = 0.11			
			day x sleep	P < 0.01			
		2-way ANOVA between subject	sex x sleep	P = 0.09	sleep	P < 0.01	C vs SD male P < 0.01;<br>C vs SD female P = 0.40
					sex	P = 0.10	
NOR training trial total exploration time	data not shown	3-way repeated measures ANOVA within subject	object x sex x sleep	P = 0.48	object	P = 0.99	
			object x sex	P = 0.82			
			object x sleep	P = 0.76			
		2-way ANOVA between subject	sex x sleep	P = 0.22	sleep	0.33	
					sex	0.05	
NOR testing trial total exploration time	3C	3-way repeated measures ANOVA within subject	object x sex x sleep	P = 0.60			
			object x sex	P = 0.10			
			object x sleep	P = 0.903	object	P < 0.0001	C male P < 0.01;<br>C female P < 0.01
		2-way ANOVA between subject	sex x sleep	P = 0.855	sleep	P < 0.05	
					sex	P = 0.45	
NOR discrimination index	3D	2-way ANOVA	sex x sleep	P = 0.75	sleep	P < 0.0001	C vs SD male P < 0.05;<br>C vs SD female P < 0.05
					sex	P = 0.87	
NOR distance travelled	3E	2-way ANOVA	sex x sleep	P < 0.01	sleep	P < 0.05	C vs SD male P < 0.01
					sex	P < 0.0001	
NOR mean speed training	3F	2-way ANOVA	sex x sleep	P < 0.01	sleep	P < 0.05	C vs SD male P < 0.01
					sex	P < 0.0001	

Peripheral tryptophan	4A	2-way ANOVA	sex x sleep	P = 0.37	sleep	P = 0.19	
					sex	P < 0.001	male vs female P < 0.001
Peripheral kynurenine	4B	2-way ANOVA	sex x sleep	P = 0.64	sleep	P = 0.44	
					sex	P < 0.01	male vs female P < 0.05
Peripheral KYNA	4C	2-way ANOVA	sex x sleep	P = 0.86	sleep	P = 0.25	
					sex	P = 0.73	
Cortical KYNA	5A	2-way ANOVA	sex x sleep	P = 0.08	sleep	P = 0.7	
					sex	P < 0.05	SD male vs SD female P < 0.05
Cortical 3-HK	data in text	2-way ANOVA	sex x sleep	P = 0.22	sleep	P = 0.65	
					sex	P = 0.14	
Hippocampal KYNA	5B	2-way ANOVA	sex x sleep	P = 0.1	sleep	P < 0.01	C vs SD male P < 0.01
					sex	P < 0.05	SD male vs SD female P < 0.01
Peripheral tryptophan GDX	6A	3-way ANOVA	sleep x sex x surgery	P = 0.3			
			sleep x sex	P = 0.4	sleep	P = 0.16	
			sleep x surgery	P = 0.93	sex	P < 0.01	sham male vs sham female P < 0.05; sham SD male vs sham SD female P < 0.01
			sex x surgery	P < 0.01	surgery	P = 0.72	
Peripheral kynurenine GDX	6B	3-way ANOVA	sleep x sex x surgery	P = 0.59			
			sleep x sex	P = 0.39	sleep	P = 0.68	
			sleep x surgery	P = 0.35	sex	P < 0.01	
			sex x surgery	P = 0.77	surgery	P < 0.01	sham male vs sham female P < 0.05
Peripheral KYNA GDX	6C	3-way ANOVA	sleep x sex x surgery	P = 0.68			
			sleep x sex	P = 0.86	sleep	P = 0.96	
			sleep x surgery	P = 0.24	sex	P = 0.67	
			sex x surgery	P = 0.52	surgery	P < 0.05	
Hippocampal KYNA GDX	6D	3-way ANOVA	sleep x sex x surgery	P = 0.15			
			sleep x sex	P = 0.46	sleep	P < 0.01	C vs SD male P < 0.01
			sleep x surgery	P = 0.09	sex	P = 0.55	
			sex x surgery	P = 0.46	surgery	P = 0.13	

Plasma corticosterone	7A	2-way ANOVA	sex x sleep	$P = 0.12$	sleep	$P < 0.01$	C vs SD female $P < 0.01$
					sex	$P < 0.01$	SD male vs SD female $P < 0.01$
Plasma corticosterone GDX	7B	3-way ANOVA	sleep x sex x surgery	$P = 0.76$			
			sleep x sex	$P < 0.01$	sleep	$P < 0.001$	C vs SD GDX male $P < 0.05$; C vs SD sham female $P < 0.05$; C vs SD GDX female $P < 0.0001$
			sleep x surgery	$P < 0.05$	sex	$P < 0.001$	
			sex x surgery	$P = 0.20$	surgery	$P < 0.001$	

Supplemental Table 1: Summary of statistical analysis. List of abbreviations: passive avoidance paradigm (PAP), novel object recognition (NOR), kynurenic acid (KYNA), 3-hydroxykynurenine (3-HK), gonadectomy (GDX), control (C), sleep deprived (SD).
